# Multidisciplinary Approach Towards Hypertensive and Chronic Alcoholic Patient With Intracerebral Bleed

**DOI:** 10.7759/cureus.29065

**Published:** 2022-09-12

**Authors:** Nidhi A Tiwari, Vikrant G Salphale, Snehal S Samal

**Affiliations:** 1 Department of Neuro Physiotherapy, Ravi Nair Physiotherapy College ,Datta Meghe Institute of Medical Sciences, Wardha, IND; 2 Department of Neuro Physiotherapy, Ravi Nair Physiotherapy College, Datta Meghe Institute of Medical Sciences, Wardha, IND

**Keywords:** ic bleed, chronic alcoholic, hypertension, rehabilitation, physical therapy

## Abstract

Intracerebral haemorrhage, the most lethal form of stroke, accounts for almost a third of all strokes. The brain receives and expels blood through blood arteries. Veins or arteries may rupture due to trauma, improper development, or excessive pressure. Blood itself has the potential to harm brain tissue. Here, we discuss the case of a 36-year-old individual who experienced giddiness, two to three seizure episodes, and left extremity weakness. Investigation revealed an intracerebral bleed. Physiotherapy was necessary to enable the patient to carry out his everyday activities comfortably in addition to medical management. The patient's condition was improved with the help of a physiotherapy protocol.

## Introduction

Bleeding into the brain by the rupture of a blood vessel is referred to as an intracerebral haemorrhage (ICH). During an ICH, symptoms frequently emerge causing sudden deterioration of consciousness and neurological dysfunction [1]. 

The region of the haemorrhage and associated oedema determine the most frequent presentation, which is a sudden onset of localised neurological impairment. This is frequently accompanied by a drop in the patient's Glasgow coma scale-measured degree of consciousness (GCS). Headache, nausea/vomiting, seizures (both convulsive and non-convulsive), and elevated diastolic blood pressure (>110 mm Hg) are other common symptoms and indicators. Obstructive hydrocephalus, which presents as postural headaches (worse on lying flat), papilloedema, nausea, vomiting, diplopia, confusion, and a decreased level of consciousness, is brought on by the extension of the clot into the ventricles. Chronic hypertension, amyloid angiopathy, anti-coagulation, and vascular abnormalities are risk factors. The resulting brain damage is frequently divided into two categories: primary, or the damage brought on by the blood clot's initial damage to the parenchyma, and secondary, or the damage brought on by its consequences [2]. 

Epidural haemorrhage, subdural haemorrhage, subarachnoid haemorrhage, and intra-parenchymal haemorrhage are the four main kinds of intracranial bleeding. Each form of bleeding has a unique aetiology, which affects the clinical symptoms, prognosis, and consequences. The types of cerebral bleeding inform the function of the interprofessional team in treating impacted individuals [3]. 

After a stroke, early functional training is advised to facilitate recuperation. However, the ideal period for functional activity recovery is unclear. A systematic review suggested that early physical therapy might be advantageous for ICH patient rehabilitation [4].

In this instance, the patient was treated and rehabilitated after being diagnosed with an intracerebral bleed. He was directed to the department of physical therapy, where a suitable, carefully thought out treatment programme was created for the patient.

## Case presentation

A 36-year-old patient who had a history of a fall on the floor was brought to the hospital. Additionally, the patient had a history of numerous seizure events. He had also previously experienced giddiness, left-sided weakness, and mouth deviation. The patient had been taking medicine for his hypertension for the past two years. The patient had a three-year history of persistent alcoholism. He was transported to a nearby hospital for a CT scan before being transferred to a rural hospital for additional care.

Investigation

**Figure 1:**
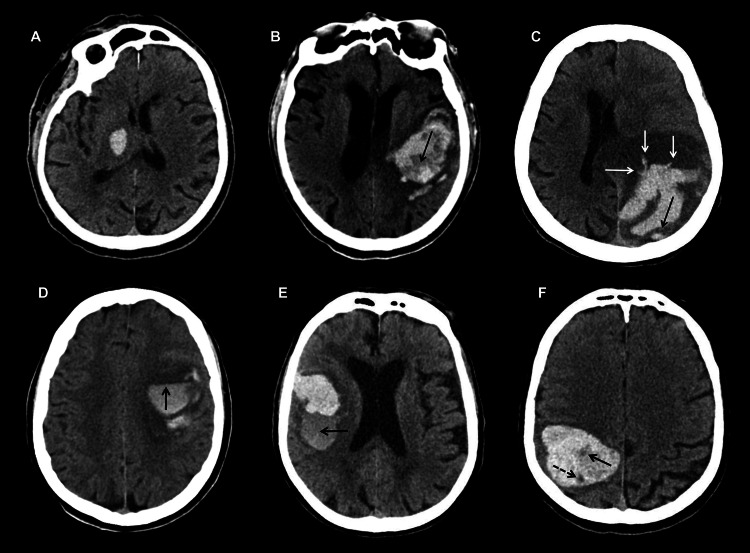
CT image showing intracerebral bleed. The lesion is causing a mass effect in the form of effacement of the right lateral ventricle adjacent sulcogyral spaces and a midline shift of 5.6 mm to left. There is an ill-defined area of hypodensity in the right frontal region. Mucosal thickening was noted in bilateral maxillary and ethmoid sinuses. Deviated nasal septum with a nasal spur to the right.

The right gangliocapsular region, right corona radiate, and right temporal region perilesional oedema of around 4.8 x 2.8 cm of the approximate volume of 45 ml are all caused by intra-parenchymal blood density collection (Figure 1).

Physiotherapy intervention

Stroke patients perform less vigorous exercise during rehabilitation (Table 1) [5]. Physiotherapy helps patients in restoring, lost skills, regain their independence, and lower their risk of further injury. In 50% of ICH patients, early progression of neurologic impairments and a decline in the level of awareness can be anticipated [6]. The patient's GCS score was 15 when he was assessed. During the examination, the patient's tone on the modified Ashworth scale was determined to be 0, for both bilateral upper and lower limbs & reflexes were normal.

**Table 1:** Therapeutic rehabilitation.

GOALS	THERAPEUTIC INTERVENTION	TREATMENT PROTOCOL
Patient education	Educating the patient on the value of exercise. Obtaining the patient's and his family's cooperation and approval.	The importance of posture, ambulation, and practical daily activities was explained to the patient and the caregiver.
To improve weakness	Ankle pumps, hamstring stretching, adductor stretch, TA stretch, static quads & hamstring.	All the intervention was given on regular basis with three repetitions and a hold of 30 seconds.
To improve strengthening	Upper limb strengthening with 1/2 lt of bottle & passive range of motion to the bilateral lower limb. Brunnstrom's technique was also used for improving strength in the hands.	All the strengthening exercise was given daily with 10 repetitions and then gradually repetition was increased.
To improve bed mobility	Rolling in bed and bedside sitting. This activity was initiated after regaining the strength of hand by the Brunnstrom approach.	The patient was shown how to roll over and sit by the bed. Brunnstrom's approach was used to increase the bed mobility of the patient.
Ambulation	Halfway ambulation was taught to the patient with the help of one or more therapist assistants. After the halfway ambulation, the patient require ambulation depending on physical assistance which he needed the continuous light touch of the therapist. After this, the patient was asked to walk under the supervision of the therapist, patient was ambulating independently over the surface and required guidance for a non-level surface. After this, the patient was independently ambulating on all the surfaces.	The patient gains confidence.

## Discussion

The most fatal type of stroke, ICH accounts for 15-30% of all strokes and is one of the most debilitating types. Veins or arteries may get rupture due to trauma, improper development, or excessive pressure. Approximately 20% of patients will regain their functional independence [7].

The most common ICH feature, a sudden onset localised neurological impairment, is determined by the location of the haemorrhage and any concomitant oedema. This is typically accompanied by a decline in the patient's level of awareness as determined by the GCS. Other typical symptoms and signs include headache, nausea/vomiting, seizures (both convulsive and non-convulsive), and elevated diastolic blood pressure (>110 mmHg). The extension of the clot into the ventricles causes obstructive hydrocephalus, which manifests as postural headaches (worse when lying flat), papilloedema, nausea, vomiting, diplopia, confusion, and a lowered level of awareness [2].

The fatal condition known as ICH has a very high fatality and morbidity rate. The other reasons why the brain bleeds include head trauma that may be brought on by a fall or by sports injury The main risk factors for ICH include hypertension and age-related amyloid angiopathy, while other risk factors include smoking, warfarin anticoagulation, excessive alcohol, and cocaine use [7]. The other reasons why the brain bleeds include head trauma that may be brought on by a fall or by a sports injury. Devices for improving walking and enabling patients to move independently, such as electromechanical and robot-assisted gait trainers, are used in rehabilitation [8]. The author in his study took 783 patients in which some patients were admitted to a physiotherapy regime therefore author concluded that patients with stroke were frequently referred for rehabilitation. Utilising inpatient physical therapy helped stroke patients stay in the hospital for shorter periods. There was little use of outpatient physiotherapy [9].

## Conclusions

Physical therapy rehabilitation has been shown to enhance patients' activities of daily living. Brunnstrom's approach was used for improving strength and the functional ambulation classification scale was used for ambulation. Patients benefit from a quicker recovery because of it. In this instance, the patient was able to carry out functional tasks on their own without concern for falling after the rehabilitation.
